# Using Vehicle-to-Vehicle Communication to Improve Traffic Safety in Sand-dust Environment

**DOI:** 10.3390/ijerph17041165

**Published:** 2020-02-12

**Authors:** Jinhua Tan, Xuqian Qin, Li Gong

**Affiliations:** School of Information and Safety Engineering, Zhongnan University of Economics and Law, Wuhan 430073, China; qinxq@zuel.edu.cn (X.Q.); gongli@zuel.edu.cn (L.G.)

**Keywords:** traffic safety, vehicle-to-vehicle communication, car-following model, sand-dust environment

## Abstract

Sand-dust environment affects drivers’ perceptions of surrounding traffic conditions, resulting in unsafe operations. From an ergonomics perspective, such adverse effects could be alleviated by environment control as well as the assistance of machines. Vehicle-to-vehicle (V2V) communication appears to be an important component of machines in future traffic systems, which could support the driving task. In order to explore what influences V2V communication would generate on traffic systems, this paper proposes a car-following model accounting for V2V communication in a sand-dust environment. The results indicate that V2V communication helps to reduce the fluctuations of acceleration, headway, and velocity, when a small perturbation is added to the traffic flow in sand-dust environment. If a vehicle in the traffic flow stops suddenly, the number of crumped vehicles decreases with V2V communication taken into account. Furthermore, the residual velocities of the crumped vehicles decrease, which means the severity of collision is suppressed. It is concluded that V2V communication can play an active role in the improvement of traffic safety in a sand-dust environment.

## 1. Introduction

According to the global status report on road safety launched by the World Health Organization (WHO) in 2018, deaths from traffic accidents have increased to approximately 1.35 million each year [1]. Moreover, the number of people suffering non-fatal injuries in traffic accidents is between 20 and 50 million, resulting in many people with disabilities [1]. Traffic accidents are a serious global public health problem [2], which happen due to many reasons [3,4,5,6]. In particular, the sand-dust environment is known as an important cause of traffic accidents. For the average deaths of each accident, sand-dust environment ranks second in adverse weather conditions, which reaches 0.455 deaths per accident [7].

From an ergonomics perspective, the traffic system is a complex man–machine–environment system [8]. The man, machine, and environment factors are all closely linked to traffic safety, where the machine factor refers to the vehicle, device, as well as related information technology [9]. In a sand-dust environment, drivers’ vision is blurred [10], which is disadvantageous to proper control of their vehicles. As a result, the risk of traffic accidents is increased. With the intent to weaken such unfavorable effects of sand-dust environment on traffic safety, some measures are taken to suppress the sand-dust from the environmental perspective [11,12]. In addition to the improvement of the environment itself, the assistance of machines is an alternative to reduce traffic risk associated with sand-dust environment. Vehicle-to-vehicle (V2V) communication is an advanced technology which appears to be an important component of machines in future traffic systems [9,13]. Under the condition of V2V communication, drivers are provided with timely information that assists them in perceiving the surrounding traffic conditions. What influences would V2V communication generate on traffic safety in a sand-dust environment? This issue is not extensively explored in the existing literature.

The car-following model, which describes the vehicle-by-vehicle following process in traffic flow, is a beneficial method to study traffic problems [14,15]. In 1961, the Newell model was put forward considering the velocity-headway relations in real traffic [16]. Numerous classical car-following models were derived on the basis of the Newell model, including the optimal velocity model (OVM) [17] and the full velocity difference model (FVDM) [18]. In 1995, Bando et al. proposed the OVM to investigate the dynamical evolution of traffic congestion [17]. Based on the OVM, Sugiyama and Nagatani studied the dynamic process of multiple-vehicle collisions when a vehicle stopped suddenly in the traffic flow [19]. In 2001, Jiang et al. proposed the FVDM, which is used to explore traffic issues such as traffic congestions and vehicle collisions [18]. On the foundation of these classical models, some extended car-following models were developed to describe traffic characteristics under different conditions [10,20]. Accounting for the impact of fog weather on drivers, Tan proposed a car-following model considering drivers’ risk illusions [20]. The analytical and numerical results indicated that the risk illusions were disadvantageous for the stability and safety of traffic flow [20]. In addition, Tan developed a car-following model with consideration of drivers’ additional reaction time due to their blurred vision in a sand-dust environment (SDM) [10]. It was found that drivers’ additional reaction time induced by sand-dust was not conducive to traffic safety [10]. Obviously, the SDM can find its potential applicability for the study of traffic flow in sand-dust environment. Moreover, to test the effects of V2V communication on traffic flow, Hua et al. [21] extended the Newell model by accounting for drivers’ timely reaction under the condition of V2V communication. The results suggested that V2V communication could improve the stability of traffic flow [21]. Evidently, the car-following model is an important tool for the evaluation of V2V communication.

In order to develop an effective management approach to improve traffic safety in sand-dust environment, this study explores the effects of V2V communication on traffic that is influenced by sand-dust. The rest of this paper is organized as follows. In Section 2, an extended car-following model is proposed, followed by a detailed introduction of linear stability analysis as well as the simulation method. The stability condition and the simulation results are described in Section 3. Section 4 presents the discussion and Section 5 draws some important conclusions.

## 2. Methodology

In this paper, accounting for the effects of V2V communication on drivers, an extended car-following model is first proposed. Then, linear stability analysis is carried out to obtain the stability criterion of the proposed model. Finally, numerical simulations are performed to explore what influences V2V communication would generate on traffic safety in sand-dust environment.

### 2.1. Model

As mentioned above, sand-dust environment blurs drivers’ vision, leading to their impaired perception of surrounding traffic conditions, which is disadvantageous to their timely response to emergencies [10]. Meanwhile, drivers have the tendency to drive at a lower velocity to avoid collision [10]. V2V communication devices could assist drivers in perceiving the surrounding traffic conditions [21]. Therefore, V2V communication has the potential to improve traffic safety by helping drivers identify traffic conditions in the sand-dust environment. Based on SDM [10], this paper proposes a car-following model in sand-dust environment incorporating the effects of V2V communication on drivers (for short, V2V-SDM). Assuming that the position of vehicle n at time t is $x_{n}(t)$, the headway between vehicle n and its preceding vehicle n+1 is $\Delta x_{n}(t)$, and $\Delta x_{n}(t)=x_{n+1}(t)-x_{n}(t)$. The dynamics equation of V2V-SDM can be formulated as:

(1) $$\frac{dx_{n}(t+T+\alpha T)}{dt}=\varepsilon V(\Delta x_{n}(t+\beta T))$$

where T depicts the delay time, which is composed of the driver’s normal reaction time and vehicle’s response time; 1/T is considered as the driver’s sensitivity; αT denotes the driver’s additional reaction time induced by their blurred vision in the sand-dust environment; ε is a parameter representing that drivers tend to drive at a lower velocity; and 0<ε<1. Parameter β is introduced to represent the pre-reaction of drivers when they are provided with information by V2V communication. The function $V(\Delta x_{n}(t+\beta T))$ refers to the optimal velocity, which can be described as follows [22]:

(2) $$V(\Delta x_{n}(t+\beta T))=V_{1}+V_{2}\tanh[C_{1}(\Delta x_{n}(t+\beta T)-l_{c})-C_{2}]$$

The parameter values in Equation (2) are $C_{1}=0.13{\;m}^{-1}$, $C_{2}=1.57$, $V_{1}=6.75\;m/s$, and $V_{2}=7.91\;m/s$ [22]. Parameter $l_{c}$ represents the length of a vehicle, and $l_{c}=5\;m$ [18,20,21,23].

In order to conduct further analysis, Equation (1) is reformulated in the form of an acceleration equation. By applying Taylor expansion to the left side of Equation (1), Equation (3) is obtained.

(3) $$\frac{dx_{n}(t+T+\alpha T)}{dt}=\frac{dx_{n}(t)}{dt}+(T+\alpha T)\frac{d^{2}x_{n}(t)}{dt^{2}}$$

Similarly, $\Delta x_{n}(t+\beta T)$ and $V(\Delta x_{n}(t+\beta T))$ in Equation (1) are expanded using the same method, as shown in Equations (4) and (5).

(4) $$\Delta x_{n}(t+\beta T)=\Delta x_{n}(t)+\beta T\frac{d\Delta x_{n}(t)}{dt}+\frac{\beta^{2}T^{2}}{2}\frac{d^{2}\Delta x_{n}(t)}{dt^{2}}$$

(5) $$V(\Delta x_{n}(t+\beta T))=V(\Delta x_{n}(t))+\beta TV^{\prime }(\Delta x_{n}(t))\frac{d\Delta x_{n}(t)}{dt}+\frac{\beta^{2}T^{2}V^{\prime }(\Delta x_{n}(t))}{2}\frac{d^{2}\Delta x_{n}(t)}{dt^{2}}$$

Inserting Equations (3) and (5) into Equation (1), we get the equation as follows:

(6) $$\frac{dx_{n}(t)}{dt}+(T+\alpha T)\frac{d^{2}x_{n}(t)}{dt^{2}}=\varepsilon V(\Delta x_{n}(t))+\varepsilon \beta TV^{\prime }(\Delta x_{n}(t))\frac{d\Delta x_{n}(t)}{dt}+\frac{\varepsilon \beta^{2}T^{2}V^{\prime }(\Delta x_{n}(t))}{2}\frac{d^{2}\Delta x_{n}(t)}{dt^{2}}$$

Therefore, the V2V-SDM can be rewritten as Equation (7).

(7) $$\frac{d^{2}x_{n}(t)}{dt^{2}}=\alpha^{\prime }\left(\varepsilon V(\Delta x_{n}(t))-\frac{dx_{n}(t)}{dt}\right)+\beta^{\prime }\frac{d\Delta x_{n}(t)}{dt}+\lambda^{\prime }\frac{d^{2}x_{n+1}(t)}{dt^{2}}$$

The functions of $\alpha {}^{\prime }$, $\beta {}^{\prime }$, and $\lambda {}^{\prime }$ in V2V-SDM are as follows:

(8) $$\alpha^{\prime }=\frac{2}{2(1+\alpha )+\varepsilon \beta^{2}TV^{\prime }(\Delta x_{n}(t))}\frac{1}{T}$$

(9) $$\beta^{\prime }=\frac{2\varepsilon \beta V^{\prime }(\Delta x_{n}(t))}{2(1+\alpha )+\varepsilon \beta^{2}TV^{\prime }(\Delta x_{n}(t))}$$

(10) $$\lambda^{\prime }=\frac{\varepsilon \beta^{2}TV^{\prime }(\Delta x_{n}(t))}{2(1+\alpha )+\varepsilon \beta^{2}TV^{\prime }(\Delta x_{n}(t))}$$

### 2.2. Linear Stability Analysis

This paper applies the perturbation method [20,21] to test the linear stability of V2V-SDM. The detailed analysis steps are as follows.

Assume that, in the initial state, *N* vehicles move on the road with a constant headway b and velocity V(b). The initial position of vehicle n at time t is

(11) $$x_{n}^{0}(t)=bn+V(b)t$$

Suppose that perturbation $y_{n}(t)$ is added to the uniform traffic flow. Then the position of vehicle n at time t becomes

(12) $$x_{n}(t)=bn+V(b)t+y_{n}(t)$$

The headway between vehicle n and the preceding vehicle n+1 at time t is calculated as

(13) $$\Delta x_{n}(t)=\Delta y_{n}(t)+b$$

Inserting Equations (11)–(13) into Equation (7), we obtain

(14) $$\begin{array}{r} \frac{d^{2}(bn+V(b)t+y_{n}(t))}{dt^{2}}=\alpha^{\prime }\left(\varepsilon V(\Delta y_{n}(t)+b)-\frac{d(bn+V(b)t+y_{n}(t))}{dt}\right)+\;\\ \beta^{\prime }\frac{d(\Delta y_{n}(t)+b)}{dt}+\lambda^{\prime }\frac{d^{2}(b(n+1)+V(b)t+y_{n+1}(t))}{dt^{2}} \end{array}$$

Further, Equation (14) can be simplified as Equation (15).

(15) $$\frac{d^{2}y_{n}(t)}{dt^{2}}=\alpha^{\prime }(\varepsilon V(\Delta y_{n}(t)+b)-V(b)-\frac{dy_{n}(t)}{dt})+\beta^{\prime }\frac{d\Delta y_{n}(t)}{dt}+\lambda^{\prime }\frac{d^{2}y_{n+1}(t)}{dt^{2}}$$

Applying Taylor expansion to $V(\Delta y_{n}(t)+b)$ in Equation (15), we have

(16) $$\frac{d^{2}y_{n}(t)}{dt^{2}}=\alpha^{\prime }(\varepsilon V(b)+\varepsilon V^{\prime }(b)\Delta y_{n}(t)-V(b)-\frac{dy_{n}(t)}{dt})+\beta^{\prime }\frac{d\Delta y_{n}(t)}{dt}+\lambda^{\prime }\frac{d^{2}y_{n+1}(t)}{dt^{2}}$$

Assuming that $y_{n}(t)=e^{ikn+zt}$ [21], we obtain the following equation

(17) $$z^{2}e^{ikn+zt}=\alpha^{\prime }(\varepsilon V^{\prime }(b)e^{ikn+zt}(e^{ik}-1)-ze^{ikn+zt})+\beta^{\prime }ze^{ikn+zt}(e^{ik}-1)+\lambda^{\prime }z^{2}e^{ikn+zt}e^{ik}$$

Then setting $z=z_{1}(ik)+z_{2}{\left(ik\right)}^{2}$ [21], Equation (18) is derived.

(18) $$-z_{1}^{2}k^{2}+z_{2}^{2}k^{4}-2z_{1}(ik)z_{2}k^{2}=\alpha^{\prime }(\varepsilon V^{\prime }(b)(e^{ik}-1)-z)+\beta^{\prime }z(e^{ik}-1)+\lambda^{\prime }z^{2}e^{ik}$$

Substituting the Euler formula $e^{ik}=\cos k+i\sin k$ into Equation (18), we get $z_{1}$ and $z_{2}$:

(19) $$\{\begin{array}{l} z_{1}=\varepsilon V^{\prime }(b)\\ z_{2}=V^{\prime }(b)\left[\frac{\varepsilon^{2}V^{\prime }(b)(\lambda^{\prime }-1)+\varepsilon \beta^{\prime }}{\alpha^{\prime }}+\frac{\varepsilon }{2}\right] \end{array}$$

When $z_{2}>0$, traffic flow is stable, and the linear stability criterion is deduced [21].

### 2.3. Numerical Simulations

To verify the theoretical results and further explore the effects of V2V communication on traffic in sand-dust environment, numerical simulations were conducted using MATLAB R2016a [24]. In the simulations, the boundary condition is supposed to be periodic [18]. The initial condition is that N vehicles are distributed on the roadway with length L=1500 m. Two simulation experiments are discussed in this paper. One is carried out to simulate the situation when a small perturbation is inserted into the traffic flow. The other considers the case when a vehicle in the traffic flow stops abruptly.

#### 2.3.1. Simulating the Effects of a Small Perturbation

Small perturbations affect the evolution processes of traffic flow, which may lead to traffic congestion and accidents [17]. In order to explore the effects of V2V communication on traffic flow, the evolution processes of vehicles’ acceleration, headway, and velocity are simulated by inserting a small perturbation into the uniform traffic flow. Parameters are set to N=100, α=0.2, ε=0.8, initial velocity $v_{ini}=30\;\mathrm{km}/h$, and the time step is 0.1 s [10,23,25]. The small perturbation added to the uniform traffic flow is the headway deviation $\Delta x_{1}(0)=2\;m$ in this study.

#### 2.3.2. Simulating the Effects of a Suddenly Stopped Vehicle

In real life, the sudden stop of a vehicle often leads to rear-end collisions [19,26]. This paper conducts simulations to test whether V2V communications reduce such adverse effects of a sudden stop in sand-dust environment. Specifically, two sub-experiments were carried out: (i) to investigate the influences on the following vehicles near the stopped vehicle and (ii) to analyze the effects on the following vehicles far away from the stopped vehicle. Here, the paper supposes that the first vehicle (n=1) stops at t=0 [19,26].

According to the collision criterion proposed by Sugiyama and Nagatani, when a vehicle approaches the preceding vehicle with a residual velocity, it will collide with the preceding vehicle [19]. In this paper, the vehicle length is supposed to be 5 m [18,20,21,23]. Correspondingly, the headway converging to 5 m implies that the vehicle might come into collision with that in front.

When a vehicle in the traffic flow stops abruptly, the first following vehicle would brake at once after the driver recognizes the emergency. However, the vehicle may be unable to stop in time and thus collides with the suddenly stopped vehicle. Similarly, for the vehicles near the suddenly stopped vehicle, the collision risk is also high. Therefore, sub-experiment (i) is conducted to investigate whether V2V communication helps to suppress the rear-end collisions in sand-dust environment.

For the vehicles far away from the stopped vehicle, they may be influenced indirectly since the braking wave propagates backwards along the platoon. Hence, sub-experiment (ii) is performed to test whether drivers far away from the stopped vehicle can make more favorable driving operations with the assistance of V2V communication. Under the periodic boundary condition, vehicle n follows vehicle n+1. Thus, the vehicle farthest from the stopped vehicle (n=1) is numbered n=2. The velocity trajectories of vehicles numbered n=2, 3, 4, 5, 6 are simulated.

## 3. Results

### 3.1. Stability Criterion

Through linear stability analysis, the linear stability criterion is given by

(20) $$V^{\prime }(b)\left[\frac{\varepsilon^{2}V^{\prime }(b)(\lambda^{\prime }-1)+\varepsilon \beta^{\prime }}{\alpha^{\prime }}+\frac{\varepsilon }{2}\right]>0$$

Further, the neutral stability condition of V2V-SDM can be obtained:

(21) $$\frac{1}{T}=2\varepsilon V^{\prime }(b)(1+\alpha -\beta )$$

When β=0, this condition is equivalent to that of SDM [10].

**Figure 1:**
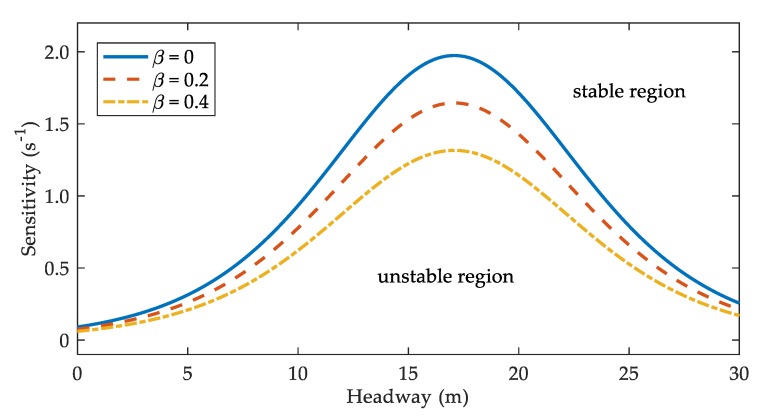
The neutral stability curves under different β.

In real life, drivers’ pre-reaction (β) may be affected by their proficiency in the use of V2V communication devices. Correspondingly, β could take different values in the theoretical analysis. Figure 1 illustrates the neutral stability curves for V2V-SDM under different β when α=0.2 and ε=0.8 [10]. The areas above and below the curves are stable and unstable regions, respectively. It is clear that the stable region is the smallest for SDM (β=0). As β increases, the stable region is enlarged. Therefore, V2V communication enhances the stability of traffic flow in sand-dust environment. Furthermore, drivers who are more experienced in the use of V2V communication devices seem to play a more active role in strengthening the stability of the traffic system.

### 3.2. Simulation Results

#### 3.2.1. Effects of V2V Communication on Traffic Flow When a Small Perturbation Is Inserted

**Figure 2:**
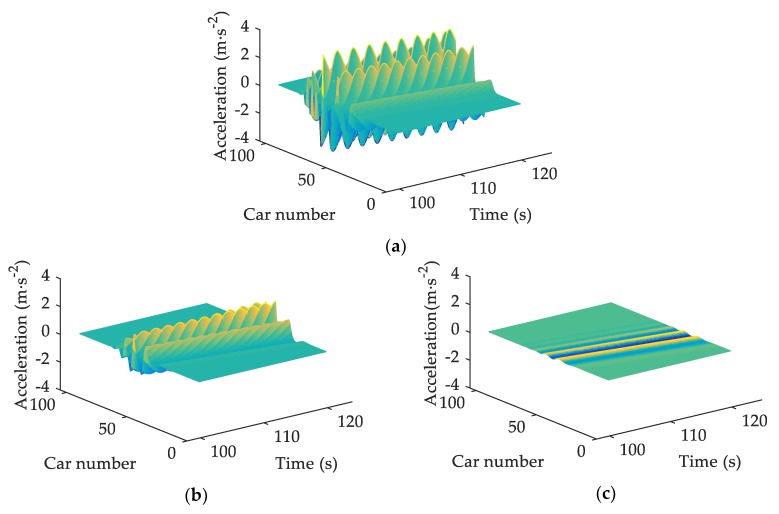
The space–time evolutions of acceleration under different β: (**a**) β=0; (**b**) β=0.2; (**c**) β=0.4, where α=0.2, T=1.2 s.

The acceleration fluctuations, to some extent, reflect the situation when a vehicle brakes or accelerates sharply. Such a phenomenon exists widely in traffic systems and is not beneficial to traffic stability [17]. Figure 2 plots the space–time evolutions of acceleration under different β. The accelerations fluctuate severely in Figure 2a, suggesting that, without consideration of V2V communication, drivers may brake or accelerate violently due to the blurred vision in sand-dust environment. In Figure 2b, the acceleration fluctuations are reduced thanks to the information from V2V communication. Comparing Figure 2a–c, it is explicit that the greater the value of β, the smaller the amplitudes of acceleration. Namely, V2V communication is conducive to traffic stability, which is consistent with the stability analysis results.

**Figure 3:**
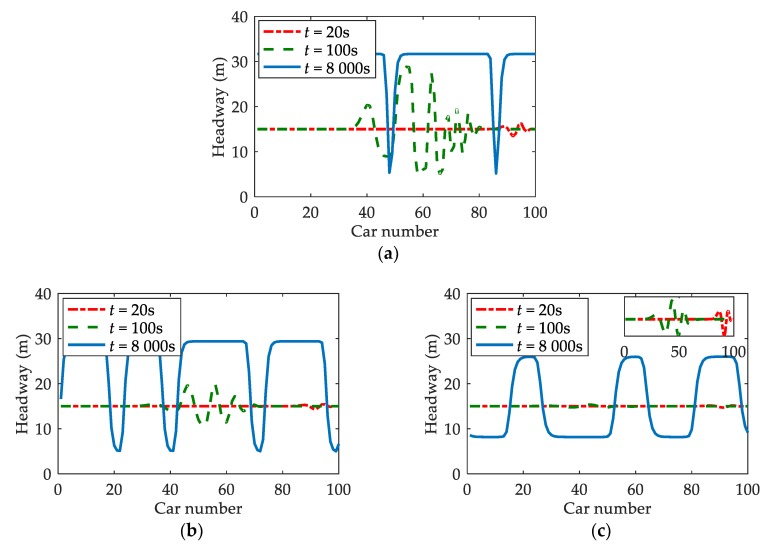
The headway evolutions under different β: (**a**) β=0; (**b**) β=0.4; (**c**) when t=20 s, t=100 s, and t=8000 s, where α=0.2, T=1.2 s.

Headway is also an important indicator for traffic stability and safety. The headway fluctuation means there may be traffic congestions [25]. At the same time, headway that is too short is not favorable to traffic safety [19,27,28]. Figure 3 shows the headway evolutions of vehicles under different β when t=20 s, t=100 s, and t=8000 s [10,20]. Comparing the three cases in Figure 3, it is obvious that the headway fluctuations become smaller with β increasing at any moment. One possible explanation is that drivers are able to adjust their driving operations in a timelier manner when they are provided with information by V2V communication. In addition, the shortest headway for vehicles in Figure 3c is always larger than that in Figure 3a,b. This demonstrates that the probability of traffic congestions or accidents can be reduced with the assistance of V2V communication.

**Figure 4:**
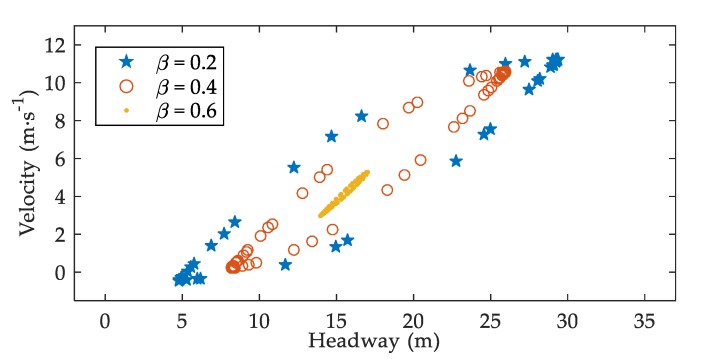
Snapshots of velocity against headway under different β when t=8000 s, where α=0.2, T=1.2 s.

Figure 4 displays the snapshots of velocity against headway when t=8000 s. One can observe that the snapshots approximately form loops with different sizes. Moreover, the size of the loop becomes smaller as β increases, which means that the amplitudes of headway and velocity are smaller. The result agrees well with the analytical results.

#### 3.2.2. Effects of V2V Communication on Traffic Flow When a Vehicle Stops Suddenly

As a vehicle in the traffic flow stops suddenly, it will exert an effect on both the vehicles following immediately behind and vehicles far away. Some important results about the effects of V2V communication on vehicles are presented as follows. In the simulations, all vehicles move with the same initial velocity $v_{ini}=30\;\mathrm{km}/h$, and the time interval is Δt=0.001 s [29].

• Influence on the vehicles near the stopped vehicle

**Figure 5:**
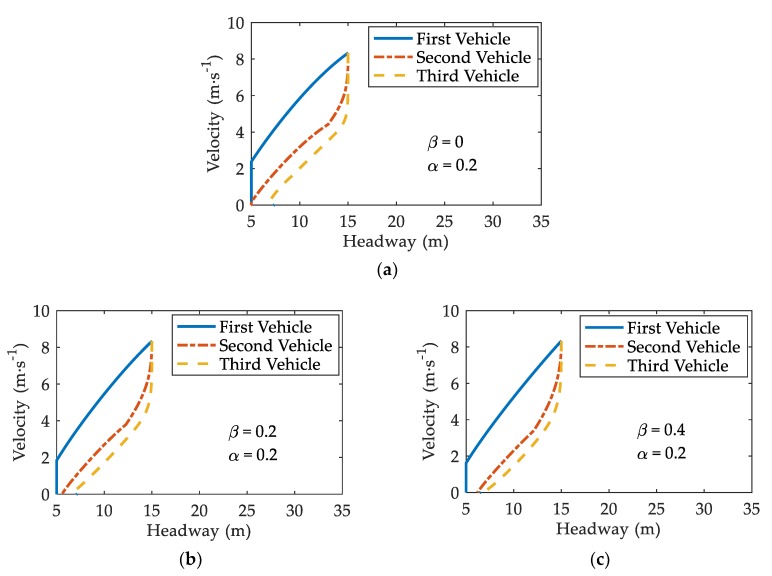
Three trajectories for three vehicles behind the suddenly stopped vehicle under different β: (**a**) β=0; (**b**) β=0.2; (**c**) β=0.4, where α=0.2, T=1.2 s.

Figure 5 depicts the trajectories for the first, second, and third vehicles behind the stopped vehicle under different β. Patterns in Figure 5a–c represent β=0, β=0.2, and β=0.4, respectively. Other parameters are set to N=100, α=0.2, ε=0.8, and T=1.2 s [10].

From Figure 5a, it is found that two vehicles come into collision with that in front when β=0. However, when β=0.2 or β=0.4, the second vehicle stops with a finite headway and does not collide with the first vehicle. Thus, V2V communication reduces the possibility of a rear-end collision in sand-dust environment. Furthermore, V2V communication plays an active role in mitigating the severity of the collision. The residual velocity of the first following vehicle in Figure 5c is lower than that in Figure 5b when colliding with the stopped vehicle. Lower velocity alleviates the severity of the collision [30]. Hence, V2V communication reduces the severity of a rear-end collision in sand-dust environment.

**Figure 6:**
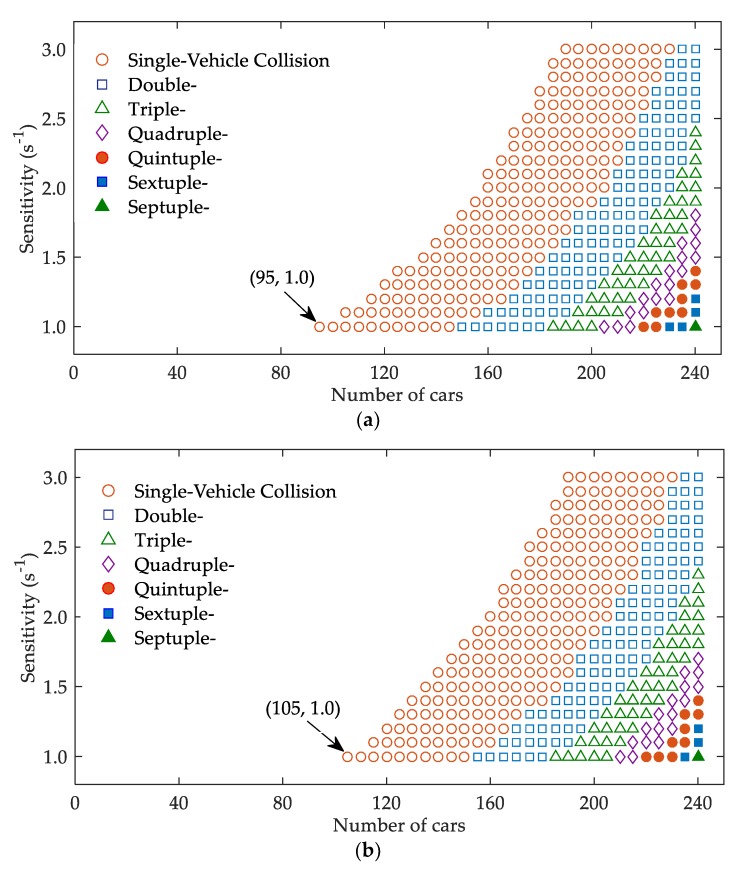
Region maps (phase diagrams) under different β: (**a**) β=0; (**b**) β=0.4.

In order to further investigate the effects of V2V communication on the possibility and severity of collision in sand-dust environment, the region maps (phase diagrams) are plotted by varying drivers’ sensitivity (1/T) and the number of vehicles (N). As shown in Figure 6, the region maps are displayed for cases (a) β=0 and (b) β=0.4 when α=0.2 and ε=0.6. The markers in Figure 6 denote the number of crumpled vehicles. The open circle, square, triangle, and diamond refer to the single-vehicle, double-vehicle, triple-vehicle, and quadruple-vehicle collisions, respectively. The full circle, square, and triangle represent the quintuple-vehicle, sextuple-vehicle, and septuple-vehicle collisions, respectively. The non-marked region describes no collision. According to the collision criterion mentioned in Section 2, when the headway of a following vehicle reduces to 5 m and its residual velocity is higher than zero, a collision occurs [19]. In this case, the vehicle is forced to stop in the simulation.

It is acknowledged that sand-dust environment will result in drivers’ decreased sensitivity [10]. Figure 6 suggests that the number of crumpled vehicles would increase as drivers’ sensitivity decreases. Thus, sand-dust environment leads to an increased number of rear-end collisions. Comparing Figure 6a,b, it is clear that the collision region shrinks as β increases from β=0 to β=0.4. Hence, V2V communication reduces the number of crumpled vehicles in sand-dust environment. Additionally, there are some special points where the numbers of crumpled vehicles do not change with the increase of β. To show the effects of V2V communication on traffic safety under these situations, Table 1 lists the residual velocities of some vehicles when they collide with their preceding vehicles. When β increases from β=0 to β=0.4, the residual velocities of the crumpled vehicles decrease. Namely, V2V communication suppresses the severity of collision in sand-dust environment.

• Influence on the vehicles far away from the stopped vehicle

**Figure 7:**
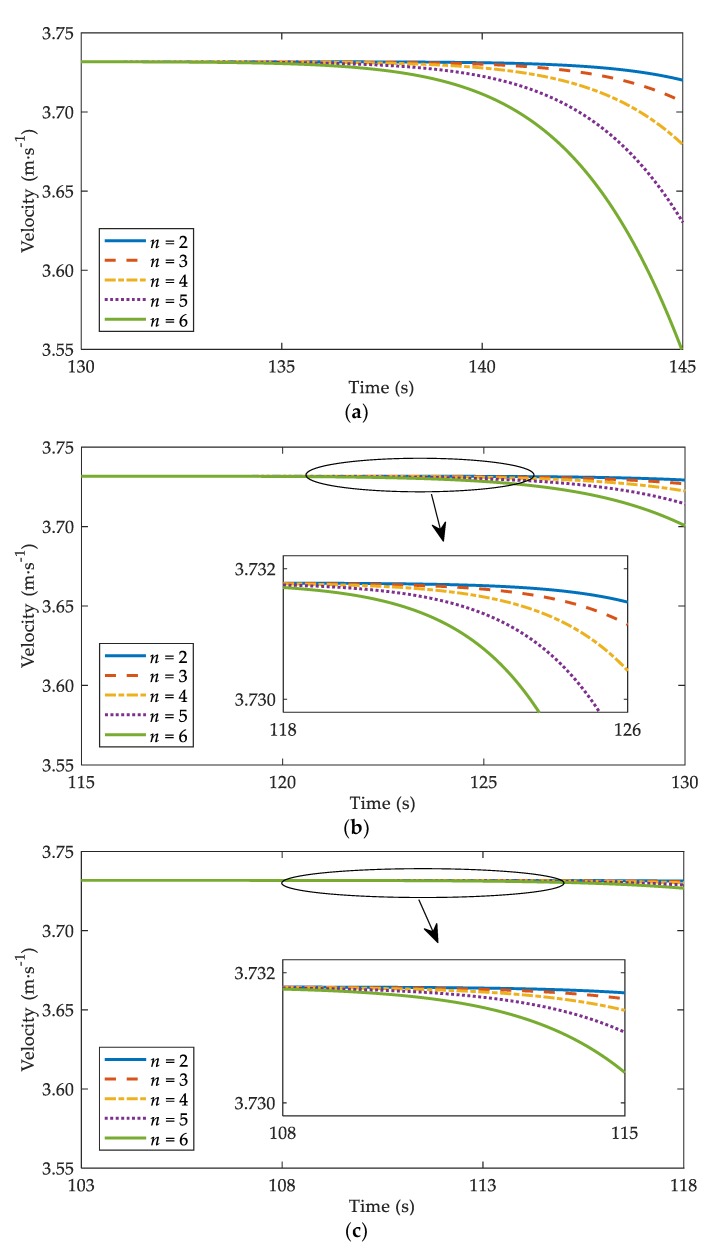
Velocity trajectories for vehicles numbered n=2, 3, 4, 5, 6 under different β: (**a**) β=0; (**b**) β=0.2; (**c**) β=0.4, where α=0.2, T=1.2 s.

Figure 7 depicts the velocity trajectories for vehicles numbered n=2, 3, 4, 5, 6 after the first vehicle (n=1) stops, where N=100, α=0.2, ε=0.8, and T=1.2 s [10]. From Figure 7, it is explicit that vehicles slow down gradually as time goes on. Comparing Figure 7a–c, one can observe that the moment vehicles start to decelerate varies with different values of β. Table 2 records the moment when vehicles numbered n=2, 3, 4, 5, 6 start to decelerate. It is clear that vehicles start to decelerate earlier under a greater value of β. Namely, with consideration of V2V communication, drivers can take timelier deceleration measures, which is conducive to traffic safety.

## 4. Discussion

Sand-dust environment is demonstrated to have considerable adverse effects on traffic safety [2,7,10]. From the standpoint of ergonomics, measures to alleviate the adverse effects of sand-dust environment can be taken from both the environmental perspective and machine perspective. Instead of controlling the environment itself, the topic of many previous studies [11,12], this paper focuses on the effects of V2V communication (machine factor) on traffic safety in sand-dust environment. Based on SDM proposed by Tan [10], the V2V-SDM is proposed in this study, which introduces the effects of V2V communications on drivers. On the basis of V2V-SDM, analytical and numerical methods are applied to explore the influences that V2V communication would generate on traffic safety in sand-dust environment.

Through linear stability analysis, the stability condition of V2V-SDM is deduced in this paper. Compared with the stable region of SDM [10], the stable region of V2V-SDM is evidently enlarged. It indicates that the linear stability is enhanced with consideration to V2V communication in sand-dust environment. Moreover, as the value of β increases, the stability is further improved.

According to simulation experiments in the literature, two kinds of perturbations are widely used for the analysis of traffic flow characteristics [19,20,21,31,32,33,34,35]. On the one hand, a small perturbation can be imposed on traffic flow to study traffic stability, which is directly related to traffic safety [20,32]. On the other hand, the perturbation caused by a sudden stop can be used to investigate multiple-vehicle collisions in traffic [19,26]. In the simulations of this paper, the effects of V2V communication on traffic safety in sand-dust environment were explored by inserting these two kinds of perturbations respectively.

Small perturbations would lead to fluctuations in acceleration, headway, and velocity of traffic flow [25,36]. By numerical simulations based on SDM, Tan proved that the fluctuations of these traffic factors would increase due to drivers’ reaction delay in sand-dust environment [10]. This paper, on the basis of V2V-SDM, simulates the evolution processes of acceleration, headway, as well as velocity by adding a small perturbation to the uniform traffic flow. The results show that the fluctuations of acceleration, headway, and velocity are all diminished when drivers are provided with information by V2V communication. It is acknowledged that, the smaller the fluctuations, the more stable the traffic flow [10,25,36]. Hence, V2V communication can enhance traffic stability in sand-dust environment. This is in line with the analytical results. Furthermore, the smaller acceleration fluctuations (Figure 2) mean that drivers’ dangerous behaviors, such as suddenly braking, are reduced [25], which is conducive to traffic safety. The smaller fluctuations of headway and velocity (Figure 3 and Figure 4), to some extent, also imply that traffic safety is improved [36]. Consequently, when a small perturbation is inserted into the uniform flow, the negative effects of sand-dust environment on traffic safety can be weakened with the assistance of V2V communication.

In order to study the impacts of V2V communication on multiple-vehicle collisions in sand-dust environment, we carried out simulations for the case that a vehicle in traffic flow stops abruptly [19,26]. The results illustrate that the number of vehicles involved in the multiple-vehicle collision has a negative correlation with drivers’ sensitivity, which is consistent with Li and Chen [26]. Moreover, the number of crumpled vehicles drops when V2V communication is used to provide traffic information (Figure 5). In Figure 6, the collision region shrinks with consideration of V2V communication, which means the possibility of a rear-end collision reduces. Furthermore, with the assistance of V2V communication, the residual velocities of crumpled vehicles decrease when collisions occur (Figure 7 and Table 1). Lower velocity can reduce the severity of the collision [30]. As a result, with the support of V2V communication, the collision severity can be suppressed as well. Consequently, V2V communication plays an active role in the risk reduction of multiple-vehicle collisions in sand-dust environment.

Parameter β takes different values in the simulations because drivers’ pre-reaction time may be different in real life. The results suggest that the value of β has an important effect on traffic safety. Particularly, a greater value of β is more conducive to traffic safety. In real traffic, to improve traffic safety, how can we increase the value of β? In other words, how can we exert an effect on drivers’ pre-reaction? There may be two major approaches. The first is to select a more favorable way to provide drivers with real-time information by V2V communication. As is known, information can be presented in the form of a sign, sound, etc. [37,38]. Different forms may have different effects on drivers [39]. Thus, it is of great importance to provide information in the most attractive form. The second is to enhance drivers’ proficiency in using V2V communication devices. Specifically, when drivers are experienced in the use of V2V communication devices, it may be easier for them to capture the critical information provided by V2V communication. In this case, drivers can respond to emergencies in a timelier fashion and their driving tasks are better supported.

## 5. Conclusions

This paper explores the management approach to improve traffic safety in sand-dust environment using V2V communication. Through linear stability analysis and numerical simulation based on the proposed V2V-SDM, the following conclusions are drawn:

(1) When a small perturbation is imposed on the traffic flow, drivers influenced by sand-dust environment will brake or accelerate violently due to their impaired perception. As a result, the stability as well as the safety of traffic system decrease. However, with information from V2V communication, drivers perceive traffic conditions earlier and adjust their driving behaviors more quickly. The fluctuations of acceleration, headway, and velocity are diminished thanks to the pre-reaction, which helps to improve traffic safety.
(2) When a vehicle in the traffic flow stops suddenly in sand-dust environment, the number of crumped vehicles decreases with the assistance of V2V communication. Meanwhile, the residual velocities of the crumped vehicles decrease when the collisions occur. The results indicate that V2V communication is able to reduce the possibility and severity of rear-end collisions in sand-dust environment. As for vehicles far away from the stopped vehicle, the moment drivers start to decelerate becomes earlier as they are provided with information by V2V communication, which is conducive to traffic safety.

The findings in this paper illustrate that V2V communication can be applied to improve traffic safety in sand-dust environment. Nevertheless, the utility of V2V communication is also affected by the drivers. Therefore, it is of great importance to look for a favorable form in the application of V2V communication and enhance drivers’ proficiency in using V2V communication devices.

## Figures and Tables

**Table 1:** The residual velocities of crumpled vehicles when the collisions occur.

Parameter Sets (N,1/T)	Crumpled Vehicles	$\mathrm{Residual}\;\mathrm{Velocity}\;(m\cdot s^{-1})$		Reduction Percentage of Residual Velocity (%)
		*β* = 0	*β* = 0.4	
(160, 1.6)	First vehicle	2.421	2.223	8.178
(160, 2.0)	First vehicle	0.797	0.558	29.987
(180, 2.5)	First vehicle	1.083	0.930	14.127
(180, 1.4)	First vehicle	4.373	4.241	3.019
	Second vehicle	0.273	0.157	42.491
(200, 1.8)	First vehicle	4.481	4.390	2.031
	Second vehicle	0.439	0.293	33.257
(200, 1.2)	First vehicle	5.779	5.688	1.575
	Second vehicle	3.250	3.151	3.046
	Third vehicle	0.561	0.502	10.517
(230, 1.4)	First vehicle	6.504	6.461	0.661
	Second vehicle	4.677	4.629	1.026
	Third vehicle	2.821	2.797	0.851
	Forth vehicle	0.845	0.836	1.065

**Table 2:** The moments starting to decelerate for vehicles numbered n=2, 3, 4, 5, 6.

Vehicle Number n	The Moment Starting to Decelerate (s)			Drivers’ Pre-Reaction Time (s)	
	β = 0	β = 0.2	β = 0.4	β = 0.2	β = 0.4
2	138.7	125.9	117.1	12.8	21.6
3	137.2	124.5	115.8	12.7	21.4
4	135.7	123.1	114.4	12.6	21.3
5	134.2	121.7	113.1	12.5	21.1
6	132.7	120.3	111.9	12.4	20.8
